# Sequences within Both the 5′ UTR and Gag Are Required for Optimal *In Vivo* Packaging and Propagation of Mouse Mammary Tumor Virus (MMTV) Genomic RNA

**DOI:** 10.1371/journal.pone.0047088

**Published:** 2012-10-16

**Authors:** Farah Mustafa, Dhuha Al Amri, Farah Al Ali, Noor Al Sari, Sarah Al Suwaidi, Preethi Jayanth, Pretty S. Philips, Tahir A. Rizvi

**Affiliations:** 1 Departments of Biochemistry, Faculty of Medicine and Health Sciences (FMHS), United Arab Emirates University (UAEU), Al Ain, United Arab Emirates; 2 Microbiology & Immunology, Faculty of Medicine and Health Sciences (FMHS), United Arab Emirates University (UAEU), Al Ain, United Arab Emirates; Institut Pasteur, France

## Abstract

**Background:**

This study mapped regions of genomic RNA (gRNA) important for packaging and propagation of mouse mammary tumor virus (MMTV). MMTV is a type B betaretrovirus which preassembles intracellularly, a phenomenon distinct from retroviruses that assemble the progeny virion at cell surface just before budding such as the type C human and feline immunodeficiency viruses (HIV and FIV). Studies of FIV and Mason-Pfizer monkey virus (MPMV), a type D betaretrovirus with similar intracellular virion assembly processes as MMTV, have shown that the 5′ untranslated region (5′ UTR) and 5′ end of *gag* constitute important packaging determinants for gRNA.

**Methodology:**

Three series of MMTV transfer vectors containing incremental amounts of *gag* or 5′ UTR sequences, or incremental amounts of 5′ UTR in the presence of 400 nucleotides (nt) of *gag* were constructed to delineate the extent of 5′ sequences that may be involved in MMTV gRNA packaging. Real time PCR measured the packaging efficiency of these vector RNAs into MMTV particles generated by co-transfection of MMTV Gag/Pol, vesicular stomatitis virus envelope glycoprotein (VSV-G Env), and individual transfer vectors into human 293T cells. Transfer vector RNA propagation was monitored by measuring transduction of target HeLaT4 cells following infection with viral particles containing a hygromycin resistance gene expression cassette on the packaged RNA.

**Principal Findings:**

MMTV requires the entire 5′ UTR and a minimum of ∼120 nucleotide (nt) at the 5′ end of *gag* for not only efficient gRNA packaging but also propagation of MMTV-based transfer vector RNAs. Vector RNAs without the entire 5′ UTR were defective for both efficient packaging and propagation into target cells.

**Conclusions/Significance:**

These results reveal that the 5′ end of MMTV genome is critical for both gRNA packaging and propagation, unlike the recently delineated FIV and MPMV packaging determinants that have been shown to be of bipartite nature.

## Introduction

The flanking long terminal repeats (LTRs) of retroviruses serve as important control regions that are responsible for regulating many aspects of retroviral replication, from gene expression (promoters, enhancers, negative regulatory elements, hormone-inducible elements, and polyadenylation sites) to reverse transcription (strand switching using “Repeat (R)” regions), and integration (attachment- att sites) [1]. In addition, for at least some retroviruses, it has been shown that the R-U5/U3-R regions at the 5′ and 3′ ends of the genomic RNA (gRNA) and their adjoining regions play important roles in RNA packaging (packaging signal- psi, ψ), dimerization (dimerization initiation site-DIS), and gRNA stability and transport (such as the constitutive transport element- CTE) [1]–[8]. Successful and specific gRNA packaging/encapsidation determines the fidelity of viral genome incorporation into the progeny virions. Studies over the past two decades have revealed the importance of the 5′ untranslated region (UTR) and beginning of the *gag* gene as crucial regions for augmenting retroviral gRNA encapsidation into the assembling particle (reviewed in [4]–[8]). For some retroviruses such as the human and simian immunodeficiency viruses (HIV and SIV), the packaging signal comprises of contiguous sequences within this region [9]–[20], while for others, the packaging signal seems to have a bi-partite nature, as has been observed for feline immunodeficiency virus (FIV) and Mason-Pfizer monkey virus (MPMV) [20]–[24].

Recently, there has been an increasing interest in studying mouse mammary virus (MMTV) replication with the hope of developing MMTV-based vectors for human gene therapy [25]. This is because of the presence of hormone inducible promoters, making it an excellent candidate for tissue-specific and hormone-inducible gene therapy (reviewed in [25], [26]). Being a non-primate retrovirus, MMTV-based vectors are likely to obviate potential safety concerns (that might result from the use of primate retroviral vectors) such as cross- and co-packaging of the transfer vector RNA by related primate retroviruses as has been observed between many retroviruses ([27]–[30] and references therein). However, the recent observation of the cross-packaging abilities of MMTV with a non-human primate retrovirus, MPMV [26], highlights the need to further enhance our understanding of the primary and secondary gRNA packaging determinants among retroviruses to establish their key contributing nature during gRNA packaging and/or cross-packaging processes.

Very little is known about the packaging determinants of MMTV that allow the virus to specifically incorporate its gRNA into the virus particle from a milieu of cellular and spliced RNAs. However, early MMTV studies showed that MMTV genome containing deletion in the *envelope* gene was competent for gRNA packaging [31], while MMTV vectors in which the 5′ MMTV LTR was swapped by that from Rous sarcoma virus (RSV) LTR [32] were defective for packaging, suggesting the importance of the 5′ region of the MMTV genomic RNA during the packaging process. Recently, we have shown that MMTV gRNA sequences from the first nucleotide (nt) in R up to 400 nts into *gag* are sufficient to allow efficient MTMV gRNA packaging and propagation, revealing the general significance of this region (R-U5-UTR-GAG) to the MMTV gRNA packaging process [33].

In order to further map the minimum viral sequences important for MMTV gRNA packaging and transfer vector RNA propagation, a systematic deletion approach was employed to delineate the extent of 5′ UTR and *gag* sequences important in these processes. Using several series of MMTV sub-genomic vectors in a biologically relevant *in vivo* packaging and transduction assay, our results reveal that unlike retroviruses such as FIV [20], [21], [23] and MPMV [22], [24], but similar to HIV and SIV [9]–[19], the entire 5′ MMTV UTR is critical for both gRNA packaging and propagation. However, the 5′ UTR by itself is not sufficient and requires additional sequences, at least 120 nt of *gag*, for efficient gRNA packaging and propagation of the packaged transfer vector RNA into the infected cells.

## Results

### Experimental Approach and *in vivo* Packaging and Propagation Assay

To study MMTV RNA packaging, the newly developed *in vivo* RNA packaging and propagation assay for MMTV was employed that utilizes an MMTV *gag/pol* expression construct (JA10) for the production of viral structural and enzymatic proteins [33], a heterologous vesicular stomatitis virus glycoprotein (VSV-G) expression vector, MD.G, for the infection of target cells with pseudotyped MMTV particles [34], and MMTV sub-genomic transfer vectors serving as substrates for RNA packaging [33]. A commercially available luciferase expression plasmid, pGL3, was added to all the transfections to monitor and normalize for transfection efficiency as described previously [23]. Seventy-two hours post transfection, culture supernatants containing infectious pseudotyped virions carrying the transfer vector RNAs were harvested and a portion was used to infect human HeLaT4 cells to determine their propagation efficiency or pelleted via ultracentrifugation on a sucrose cushion for isolating packaged transfer vector RNA. If the assembling VSV-G-pseudotyped MMTV particles were able to package the transfer vector RNAs with the different deletions, successful transduction events could be scored by the appearance of hygromycin resistant colonies 10–12 days post infection upon hygromycin selection. To calculate the relative packaging efficiency for each transfer vector RNA, the amount of gRNA present in the pelleted virions was quantified by reverse transcriptase (RT) and real time PCR using β -actin as an endogenous control. Finally, the values were normalized with the luciferase expression to control for transfection efficiency.

### MMTV gRNA Packaging and Propagation Studies Using Subgenomic Transfer Vectors Containing Wild Type LTRs

**Figure 1:**
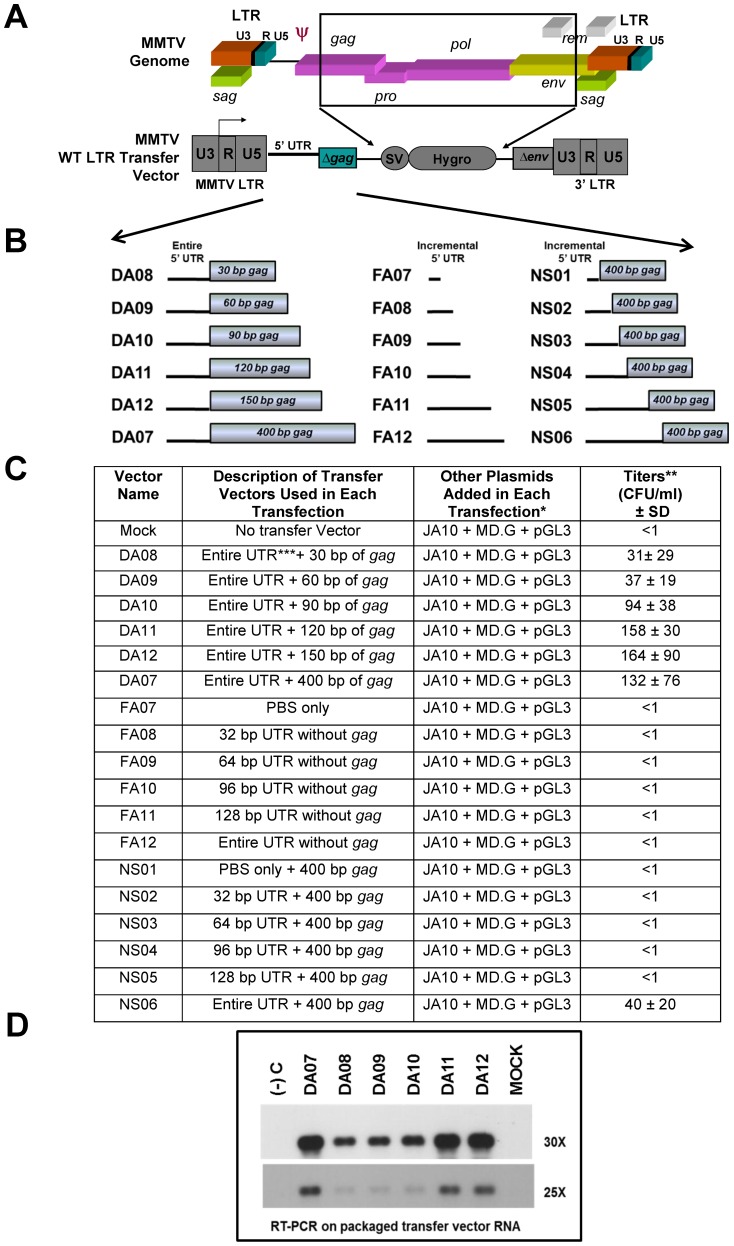
Experimental design to determine the role of the 5′ untranslated region (UTR) and *gag* sequences in MMTV RNA packaging. (**A**) Schematic representations of the complete MMTV genome and the MMTV long terminal repeats (LTRs)-based transfer vectors used as RNA packaging substrates in the study. In these transfer vectors, the wild type (WT) MMTV LTRs were maintained and therefore following transfection the RNA transcription was initiated by the promoter sequences within the U3 region of the 5′ LTR. Region encompassing most of the structural and enzymatic genes (*gag*, *pro, pol*, part of *env* and *rem*) were replaced by the SV-40 *hygromycin* resistance gene cassette as a marker for successful transduction of the target cells by packaged transfer vector RNA. (**B**) Three series of deletion mutations at the 5′ end of MMTV transfer vector sequences were introduced to monitor their effect on RNA packaging and propagation. The first series of deletion mutants (DA07–12) contained the entire 5′ UTR in the presence of incremental amounts of *gag* sequences, the second series of mutants (FA07–12) contained deletions in the 5′ untranslated region (UTR) sequences in the absence of any *gag* sequences, while the third series of mutants (NS01–06) contained the same incremental amounts of 5′ UTR sequences in the presence of 400 nucleotides (nt) of *gag*. (**C**) Table showing viral titers observed post transduction of HeLaT4 target cells by transfer vector RNAs tested. None of the transfer vector RNAs containing deletions in the 5′ UTR in the absence (FA07–FA12) or the presence (NS01–NS05) of *gag* sequences could be propagated (<1 CFU/ml) except NS06. *JA10, MMTV packaging Gag/Pol expression construct; MD.G, vesicular stomatitis virus (VSV-G) Env expression plasmid; pGL3, luciferase expression plasmid. **Propagation of the transfer vector RNA expressed as hygromycin resistance colony forming units (CFU)/ml of viral supernatant that was used to infect target cells. ***The entire UTR refers to 160 nt excluding 17 nt of primer binding site (PBS). The data represents mean of at least three independent transfection and infection experiments testing all mutants and was derived after normalization to the transfection efficiencies observed by luciferase expression from a co-transfected luciferase expression vector. SD, standard deviation. (**D**) Reverse transcriptase (RT) PCR analysis of the MMTV LTR-based DA series of transfer vectors containing incremental amounts of *gag* sequences in the presence of the entire 5′ UTR followed by Southern blotting. The probe was prepared by PCR amplification of a 142 nt long R/UR/5′ UTR region (nt 1179–1321) common to all the transfer vectors, as described in Materials and Methods. Amplification was carried out for either 25 (lower panel) or 30 cycles (upper panel) using transfer vector-specific primers OTR671 and OTR 672.

Briefly, MMTV-based retroviral vectors were created that contain the wild type 5′ MMTV LTR, a *hygromycin resistance* gene driven from the simian virus 40 (SV40) promoter as a marker for successful propagation, a truncated MMTV *env* gene, and the 3′ MMTV LTR (Figure 1A). Different permutations of the 5′ UTR and *gag* sequences were introduced into the MMTV sub-genomic vector (Figure 1B) and tested using the *in vivo* packaging and propagation assay. The first series of vectors tested the entire 160 nt of the 5′ UTR in the presence of incremental amounts of *gag* up to the first 400 nt of *gag* (Figure 1B). The second series of vectors tested incremental amounts of 5′ UTR in the absence of any *gag* sequences, while the third series tested the same incremental 5′ UTR sequences in the presence of 400 nt of the *gag* (Figure 1B).

Initial test of the wild type LTR-based MMTV vectors revealed very low transfer vector RNA propagation efficiency as measured by the transduction of the target cells with *hygromycin* resistance gene (Figure 1C). This translated to viral titers of ∼1×10^1^–2.5×10^2^ colony forming units/ml (CFU/ml) even with the vector DA07 containing the entire 5′ UTR and 400 nt of *gag* (Figure 1B). In fact, none of the transfer vector RNAs containing incremental amounts of the 5′ UTR showed any ability to be propagated (<1 CFU/ml) except NS06 with ∼40 ± 20 CFU/ml (Figure 1C).

Next, the packaging ability of the DA vector series (containing the entire 5′ UTR along with incremental amounts of *gag*) that could be propagated, although inefficiently, was tested. Reverse transcriptase (RT) PCR of the DA series of vector RNAs packaged by the virus particles revealed that the vector RNAs could be packaged, but quite inefficiently with some of the samples requiring Southern blotting for visualization of the packaged RNA (Figure 1D). Southern blotting of the resultant PCR products revealed that at least 120 nt of *gag* were required to observe efficient RNA packaging (Figure 1D), which corroborated with the RNA propagation data for these vectors (Figure 1C). The inefficient RNA packaging and propagation abilities could be attributed to the low expression of the transfer vector RNAs perhaps due to the use of wild type 5′ MMTV LTR promoter that was used for their transcription in human cells. The MMTV LTR is a well-known hormone inducible promoter that has low basal promoter activity and requires induction with glucocorticoid hormones such as dexamethasone [35], [36]. However, the gRNA packaging and propagation results obtained were following induction of the transfected cultures with 10^−6^ M dexamethasone (Figure 1C and 1D), suggesting an inherent MMTV promoter caveat while using human cells.

Besides possible promoter restrictions, the poor gRNA packaging and propagation results obtained could have been due to the inefficient export of the transfer vector RNAs out of the nucleus in the absence of an intact Rem/Rem Responsive Element (RmRE) regulatory export pathway. This pathway has recently been described in MMTV [37], [38], and was perturbed during cloning of these subgenomic vectors. Specifically, an intact RmRE is maintained; however, the Rem protein that binds to RmRE (facilitating the efficient export of unspliced MMTV gRNA) is not expressed in these transfer vectors due to a truncation in the Rem open reading frame during the cloning process (Figure 1A). Therefore, it is possible that the poor gRNA packaging and propagation results obtained could also have been in part due to the inefficient export of the transfer vector RNAs out of the nucleus in the absence of an intact Rem/RmRE regulatory export pathway.

### Improvement of Transfer Vector Design

**Figure 2:**
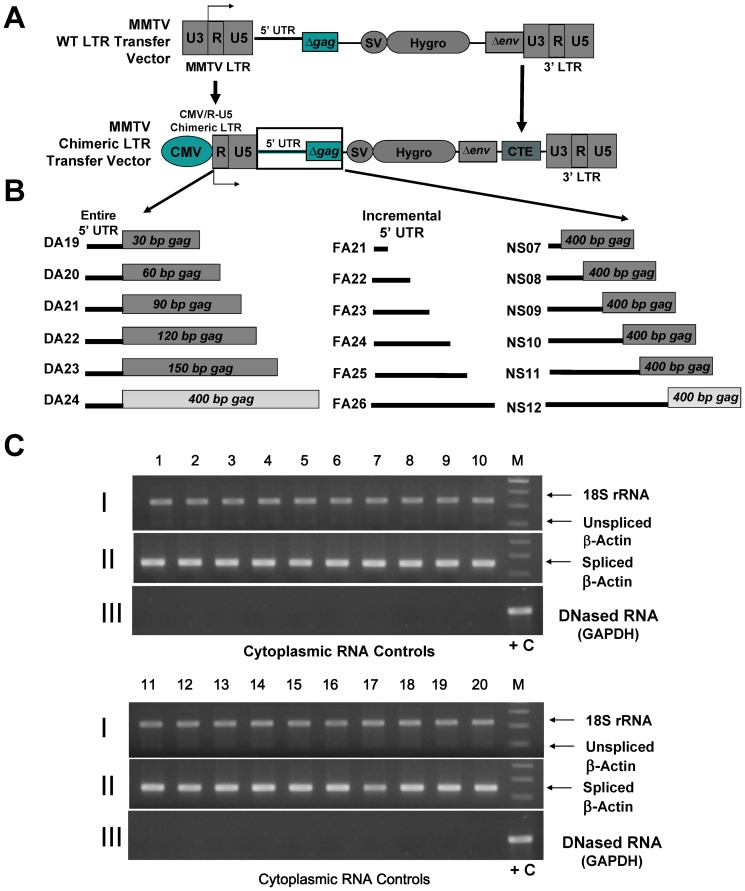
Improvement in the design of MMTV LTR-based transfer vectors and their test in the *in vivo* packaging assay. (**A**) Schematic representation of the second set of MMTV transfer vectors in which the U3 region containing the promoter within the 5′ LTR was replaced with the promoter sequences of human cytomegalovirus (hCMV) generating a chimeric CMV-R/U5 LTR. In addition, the constitutive transport element (CTE) from the Mason-Pfizer monkey virus (MPMV) was inserted between the Δ*env* and the 3′ LTR to facilitate efficient nuclear export of viral genomic RNA in the absence of an intact MMTV Rem/RmRE export pathway. (**B**) Schematic representation of the three series of transfer vectors tested in the study containing chimeric 5′ MMTV LTR and therefore following transfection the RNA transcription was initiated by the hCMV promoter sequences. The first series of transfer vectors (DA19–24) tested the entire 5′ UTR in the presence of incremental amounts of *gag* sequences, the second series (FA21–26) tested only incremental amounts of 5′ UTR sequences in the absence of any *gag* sequences, while the third series (NS07–12) tested the same incremental amounts of 5′ UTR sequences in the presence of 400 nt of *gag*. (**C**) RT-PCR of cytoplasmic RNA fractions to ensure the integrity of the fractionation technique and conventional PCR on RNA samples to monitor the absence of any contaminating DNA in the RNA preparations. Panel I: Multiplex PCR amplification of cytoplasmic cDNAs with 18S and unspliced ß-actin primers; Panel II: PCR amplification of cytoplasmic cDNAs with spliced ß-actin primers; Panel III: PCR amplification of DNase-treated RNA with GAPDH primers. Lane numbers 1–18 correspond to the numbers of the three series of chimeric LTR transfer vectors (DA, FA, and NS) in sequential order, whereas lanes 19 and 20 correspond to cytoplasmic fraction from Mock (containing the packaging construct, JA10 + the VSV-G-Env expression plasmid, MD.G + luciferase-expression vector, pGL3) and no DNA transfected cultures. +C, positive control (cDNA from cellular mRNA); M, molecular weight markers.

To circumvent any potential problems with poor MMTV promoter efficiency in human cells and RNA export defects, the 5′ MMTV LTR was modified by replacing the U3 region (containing the promoter) with the constitutively active human cytomegalovirus (hCMV) promoter that has been classically used as a high efficiency promoter in mammalian cells (Figure 2A). A similarly constructed chimeric CMV/RU5 promoter has been successfully used to express MMTV [33], [39] and FIV-based vectors [20], [21], [23], [40]–[42]. Furthermore, to ensure efficient nucleocytoplasmic transport of transfer vector RNA, the constitutive transport element (CTE) from MPMV [43] was cloned at the 3′ end of the transfer vectors between the truncated envelope sequences and the 3′ LTR (Figure 2A). The MPMV CTE has been successfully shown to work effectively in such heterologous systems for HIV [43], SIV [44] FIV [41], and MMTV [33].

### Role of MMTV 5′ UTR and *gag* Sequences in MMTV gRNA Packaging and Propagation

The modified transfer vectors (Figure 2B) were co-transfected individually along with the packaging construct, JA10 and the *env* expression vector MD.G for virus production into 293T cells. Following transfection, virus particles were isolated from each transfected cultures to quantify the effect of the introduced mutations on both gRNA packaging and propagation. However, before quantifying the viral RNA contents in the viral particles, it was imperative to determine whether each vector RNA was expressed stably and transported to the cytoplasm successfully.

#### Stable cytoplasmic expression of all transfer vector RNAs

In order to ascertain stable expression and efficient nuclear export of transfer vector RNAs, nuclear and cytoplasmic RNA fractions were prepared from the transfected cells and DNase-treated to ensure removal of any contaminating plasmid DNA (Figure 2C, panel III). This was followed by their conversion into cDNA by reverse transcriptase to allow amplification of the original transfer vector RNAs. Next the integrity of the RNA fractionation technique was tested to ensure that there was no leakage of the nuclear fraction into the cytoplasmic fraction by taking advantage of differential distribution of the unspliced and spliced β-actin mRNA message in the nuclear and cytoplasmic fractions, respectively [45] as described previously [23]. Attempts to amplify unspliced β-actin mRNA in a multiplex RT-PCR did not show its detectable amplification in the cytoplasmic fractionation (Figure 2C; panel I), in contrast to the presence of spliced β actin and 18S rRNA mRNA in the cytoplasm, as expected (Figure 2C; panels I and II), further authenticating the amplifiability of our cDNA preparations.

#### Development of a relative quantification assay using real time PCR

**Figure 3:**
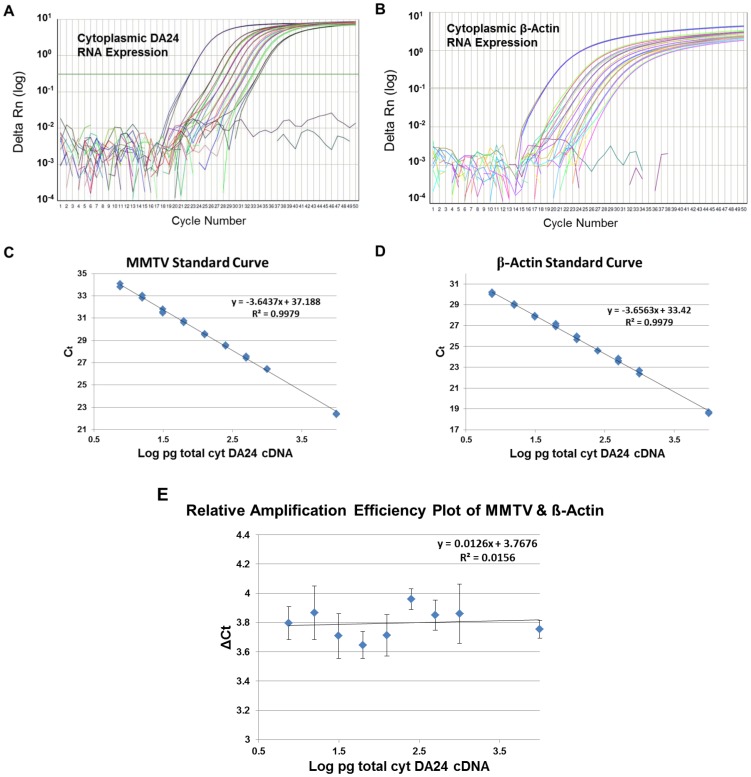
Validation of the real time PCR assay developed for relative quantification of transfer vector RNA expression using the Ct slope method. Determination of the threshold cycles (Ct) of the cDNA prepared from 293T cytoplasmic RNA expressing transfer vector DA24 tested in triplicates as 10- and 2-fold dilutions by the (**A**) custom-made MMTV Taqman assay, and (**B**) the ß-actin Taqman assay. ΔRn is the target gene-specific fluorescence signal (FAM for MMTV-specific and VIC for ß-actin-specific sequences) normalized to the signal for the internal passive control, ROX (Normalized Reporter or Rn) from which the baseline target fluorescence has been subtracted (ΔRn = Normalized Reporter (Rn) - baseline). Standard curves of the (**C**) MMTV and (**D**) ß-actin Taqman assays were generated to determine their ΔCt values (Ct values of the MMTV assay – Ct value of the ß-actin endogenous control) that were needed to allow comparison of amplification efficiencies of the two assays. (**E**) Relative amplification efficiencies of the two assays as determined by the analysis of ΔCt value variations with the amount of input template cDNAs. For the two assays to have similar amplification efficiencies, the value of the slope of log input amount verses ΔCT should be approximately zero (<0.1), which under our experimental conditions were calculated to be 0.0126, thus validating the assay for the relative quantification analysis.

The relative expression of the various transfer vector RNAs in the cytoplasm was tested using a custom-made MMTV Taqman real time PCR assay in combination with a commercially-available-endogenous β-actin Taqman assay (Applied Biosystems). The custom MMTV Taqman assay was designed within the U5 region of the transfer vector RNAs, enabling detection of all mutant transfer vector RNAs via a single primer/probe combination. To allow a relative comparison of the transfer vector RNA expression using β-actin as an endogenous control, we determined whether the amplification efficiencies of the two assays were equivalent. This was accomplished by first determining the value of the threshold cycle, Ct (Figure 3A and 3B), followed by ΔCt values (Ct values of the MMTV assay – Ct value of the ß-actin endogenous control) (Figure 3C and 3D). Finally, we analyzed how the ΔCt values varied with the amounts of input template cDNA as a sensitive indicator of relative amplification efficiencies of the two assays. The value of the slope of log input amount verses ΔCT should be approximately zero (<0.1) for the two assays to have similar amplification efficiencies (User Bulletin #2, ABI PRISM 7700 Sequence Detection System). As can be observed from Figure 3E, the slope under our experimental conditions was calculated to be 0.0126, validating the assay for the relative quantification analysis.

**Figure 4:**
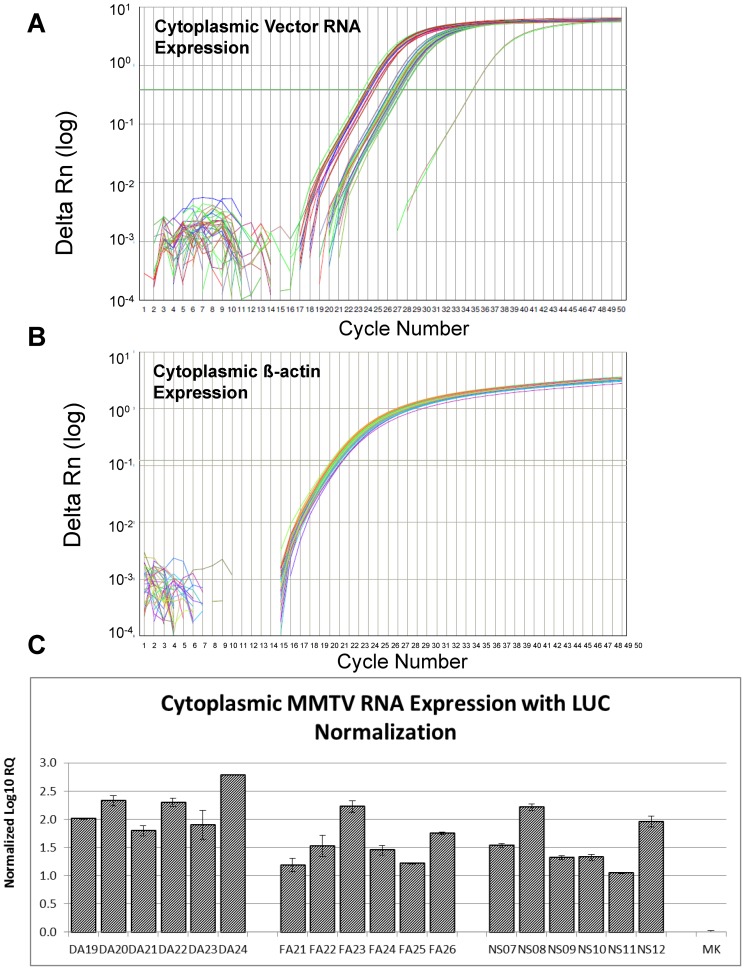
Relative expression of transfer vector RNAs within the transfected cells. Real time PCR analysis of (**A**) cytoplasmic transfer vector RNAs and (**B**) cytoplasmic β-actin RNA expression expressed as logΔRn verses cycle number. ΔRn is the target gene-specific fluorescence signal (FAM for MMTV-specific and VIC for ß-actin-specific sequences) normalized to the signal for the internal passive control, ROX (Normalized Reporter or Rn) from which the baseline target fluorescence has been subtracted (ΔRn = Normalized Reporter (Rn) - baseline). (**C**) Relative cytoplasmic transfer vector RNA expression in 293T cells after normalization with β-actin and luciferase expression. MK, Mock, transfected cultures with packaging construct, JA10 + the VSV-G-Env expression plasmid, MD.G + luciferase-expression vector, pGL3 except the transfer vector. RQ, Relative Quantification in log_10_ units. The primers for detecting the transfer vectors were designed within the U5 region of the MMTV LTR, a region common to all transfer vector RNAs. Each sample was tested in duplicates with MMTV- and β-actin-specific probes and primers as described in Materials and Methods.

Test of the cDNAs prepared from the cytoplasmic RNA fractions revealed that the transfer vector RNAs were stably expressed between Ct values of 23.5–27.5 and efficiently exported to the cytoplasm (Figure 4A). Alongside vector RNA expression in the cytoplasm, the expression of the endogenous β-actin mRNA was also monitored as a control for the amount of the input sample tested in the assay (Figure 4B). As observed, all samples expressed β-actin mRNA at very similar levels, within one Ct value of each other (Figure 4B). Normalization of the transfer vector RNA expression with the level of endogenous β-actin and exogenous luciferase expression revealed that all transfer vector RNAs were expressed in the cytoplasm within approximately 2–3 folds of each other (Figure 4C). These ancillary controls were incorporated into our experimental setup to confirm the integrity of the RNA packaging and propagation assay and to further validate that the results obtained were bonafide.

### The Entire MMTV 5′ UTR and ∼ 120 nt of *gag* are Needed for Optimal MMTV gRNA Packaging

**Figure 5:**
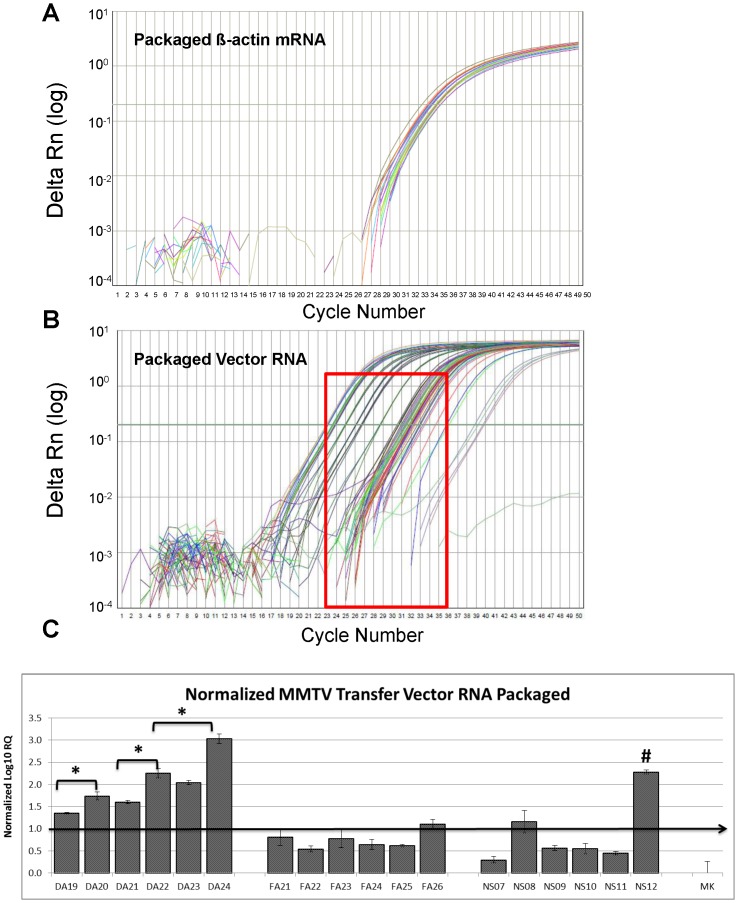
Relative packaging efficiency of MMTV transfer vector RNAs into the pseudotyped MMTV particles. Real time PCR analysis of (**A**) packaged β-actin RNA into VSV-G-Env-pseudotyped MMTV particles and (**B**) packaged transfer vector RNAs into the VSV-G-Env-pseudotyped MMTV particles expressed as ΔRn verses cycle number. ΔRn is the target gene-specific fluorescence signal (FAM for MMTV-specific and VIC for ß-actin-specific sequences) normalized to the signal for the internal passive control, ROX (Normalized Reporter or Rn) from which the baseline target fluorescence has been subtracted (ΔRn = Normalized Reporter (Rn) - baseline). (**C**) Relative RNA packaging efficiencies for each of the mutant transfer vector RNA after normalization with β-actin and luciferase expression. The box in panel B highlights the wide range of threshold cycle (Ct) values observed for each transfer vector in comparison to the similar amounts of β-actin packaged into the viral particles. The arrow in panel C highlights the threshold value of detection. MK, Mock, cells transfected with packaging construct, JA10 + the VSV-G-Env expression plasmid, MD.G + luciferase-expression vector, pGL3 except the transfer vector. RQ, Relative Quantification in log_10_ units. *, statistically significant differences between constructs are shown by the brackets (p<0.01). #, statistically significant difference between NS12 and DA24 (p<0.01). The primers/probe for detecting the transfer vectors were designed within the U5 region of the MMTV LTR, a region common to all transfer vector RNAs. Each sample was tested in triplicates with MMTV- and β-actin-specific probes and primers in panel B, while the β-actin samples shown in panel A were tested in duplicates, as described in Materials and Methods.

Next, the packaging efficiency of the transfer vector RNAs into the pseudotyped MMTV particles was assessed by the same custom-made real time PCR assay as described above. Towards this end, the first question asked was whether a cellular housekeeping β-actin mRNA could be used as an endogenous control in our assays since it is not a recognized virion-associated mRNA. It has now been well-established that cellular RNAs can be packaged into retroviral particles as has been shown for several RNAs such as 7SL RNA, U6 snRNA, tRNA, or mRNAs for cyclophilin A and for several types of ribosomal proteins, and even ribosomal RNA itself [18], [46]–[51]. Therefore, we tested whether β-actin mRNA could be packaged into the MMTV particles and at similar levels irrespective of the transfer vector packaging efficiency (Figure 5A). As observed, β-actin mRNA could indeed be detected quite consistently inside the virus particles within 2 Ct values of each other, starting from 33.5 Ct-35.5 Ct, irrespective of whether transfer vector RNAs were packaged efficiently or poorly within the virus particles (Figure 5B). This observation is consistent with the finding of Rulli and colleagues who observed that a majority of the cellular RNAs packaged in the MLV and HIV-1 virus particles were packaged non-selectively and merely represented the proportions in which they were found in the cytoplasm of the virus-producing cells [51]. Thus, this enabled the use of β-actin mRNA as an endogenous control while calculating relative packaging efficiency of the transfer vector RNAs in our custom-made real time PCR assay.

As opposed to β-actin mRNA, all transfer vector RNAs were encapsidated with widely different packaging efficiencies, encompassing raw Ct values between 23–36, depending upon the amount and type of sequence present at the 5′ end of the sub-genomic RNAs (see boxed region in Figure 5B). The DA series of vectors (DA19–DA24) were packaged between Ct values of 23 to 28, the FA series of vectors (FA21–FA26) were packaged between Ct values of 30–34, while the NS series of vectors (NS07–NS12) were packaged between Ct values of 32–34 with the exception of NS12 that was packaged at a Ct value of 24 (Figure 5B). The mock-transfected samples obtained following transfection with the Gag/Pol and VSV-G Env expression plasmids along with pGL3 luciferase expression vector but no MMTV transfer vector, showed a Ct value of 37, while the no template control showed a Ct value of 39, revealing the actual background of the assay. These values were then normalized to the amount of endogenous β-actin mRNA observed in each sample.

Further normalization of the transfer vector RNAs packaged into the virus particles was performed to take into account differences in transfection efficiency observed using the luciferase expression vector in each transfected cultures (Figure 5C). The sample from the mock transfection was used to set the threshold value of detection of RNA packaging stringently at ∼1.0 normalized log_10_ Relative Quantification (RQ) units (∼ three-folds above the mock level).

Normalization of the data revealed that the DA series of transfer vectors with the entire 5′ UTR and incremental amounts of *gag* were the most efficiently packaged (at least 3–6 folds higher on a log_10_ scale) compared to vector series with incremental amounts of 5′ UTR either without *gag* (FA series) or with *gag* (NS series) except for NS12 (Figure 5C). In fact, compared to the threshold of detection (shown by the horizontal arrow in Figure 5C), packaging of most of the FA and NS series of vectors was barely detectable except for NS12 (the vector with complete 5′ UTR and 400 nt of *gag*) that was efficiently packaged and comparable to the DA series of vectors. Overall, these results revealed that the intact 5′ UTR is critical for efficient RNA packaging but is not sufficient by itself for this crucial step of MMTV life cycle.

Within the DA series (DA19–DA24), as the incremental amount of *gag* sequences increased, so did the packaging efficiency with DA24 containing 400 nt *gag* being the vector packaged most efficiently (Figure 5C). However, there was no difference in the packaging efficiency of DA22 and DA23, vectors containing 120 or 150 nt of *gag* respectively and these vector RNAs were packaged within < one log of DA24 (p<0.01). These results corroborated well the observations made earlier using MMTV vectors harboring similar sequences and containing wild type MMTV LTR as the promoter (Figure 1) that 120 nt of *gag* are sufficient along with the entire 5′UTR to allow efficient packaging of MMTV-based vector RNAs, though inclusion of *gag* sequences upto 400 nt increased that by a third (compare Figures 1D and 5C).

Interestingly, NS12, the vector with the entire 5′ UTR and 400 nt of *gag* (comparable to DA24), showed lower packaging efficiency (by about 0.7 logs compared to DA24; p<0.01) and was similar to DA22 and DA23, vectors with 120 and 150 nt of *gag* sequences, respectively (Figures 2B and 5C). The NS vector series was cloned in a different manner and contains an *Spe*I site that was introduced between the 5′ UTR and beginning of *gag* sequences for the cloning process that is absent in the DA series. It is possible that presence of the *Spe*I site may have perturbed the higher order structure, if any, of this important region in some subtle manner, resulting in lower packaging efficiencies. The same may hold true for DA07 and NS06, although we did not directly test the packaging efficiency of NS06 vector RNA (Figure 1). Taken together, these results suggest that the entire 5′ UTR and approximately 120 nt of *gag* seem to be minimally required for efficient packaging of MMTV transfer vector RNAs.

### The 5′ MMTV UTR is Critical but not Sufficient for Efficient MMTV RNA Packaging

Analysis of the FA series of vectors (FA21–FA26 containing incremental amounts of 5′ UTR sequences in the absence of any *gag* sequences, Figure 2B) revealed that the entire 5′ UTR is not sufficient for efficient RNA packaging since the RNA of FA26 containing the entire 5′ UTR but no *gag* sequences could be barely packaged within the viral particles (Figure 5C). However, the 5′ UTR was observed to be important for RNA encapsidation since even a 32 nt deletion at the 3′ end of the 5′ UTR in the presence of 400 nt of *gag* (NS11) resulted in greater than 100-fold (∼2 log) drop in RNA packaging efficiency compared to the vector RNA with the entire 5′ UTR, NS12 (Figure 5C). Similarly, the vector with the entire 5′ UTR in the absence of *gag* (FA26) was packaged at a greater than 10-fold (1 log) lower efficiency than NS12 and greater than 60-fold (∼1.8 log) lower efficiency than DA24, vectors containing the entire 5′ UTR in the presence of 400 nt of *gag* (p<0.01), confirming that *gag* sequences are indeed required during MMTV gRNA packaging (Figure 5C). As observed with MMTV vectors with the wild type LTRs, ∼120 nt of *gag* were required for improving the RNA packaging efficiency in the presence of the entire 5′ UTR by 10-fold, as opposed to 30–90 nt of *gag* which did not enhance packaging efficiency significantly (compare FA26 with DA19–DA22 in Figure 5C and DA11 with DA08–DA10 in Figure 1D).

### The 5′ MMTV UTR is Critical but not Sufficient for MMTV Transfer Vector RNA Propagation and Requires at least 120 nt of *gag* as well

Test of the new series of transfer vectors in the propagation assay further revealed that the LTR promoter and CTE modifications resulted in several folds increase in the pseudotyped virion titers as demonstrated by an increased appearance of hygromycin resistant colonies in the infected cultures (compare Table 1 with Figure 1C). However, despite the overall increase in vector RNA propagation (DA series of vectors), most of the vectors with incremental amounts of the 5′ UTR sequences with or without *gag* (the FA and NS series, Figure 1B) could not be successfully propagated except for NS12 (Table 1). This is a very unusual finding compared to what has been observed in similar vector systems of MPMV and FIV employing a similar deletion approach [20]–[23]. The DA-series of vectors that could be propagated successfully revealed that whereas 120 nt of *gag* were sufficient for vector RNA propagation, 400 nt increased that efficiency by two-folds (Table 1). These observations revealed that, 1) the 5′ end of MMTV RNA (R-U5-UTR-*gag*) contains elements critical for proper completion of steps in vector RNA propagation, such as RNA dimerization, encapsidation, reverse transcription, and integration since our readout assay for RNA propagation was dependent on the successful completion of these crucial steps in virus life cycle, and 2) the entire 5′ UTR itself is not sufficient and requires ∼120–400 nt of *gag* for efficient vector RNA propagation. Overall, these results mimicked the RNA packaging and propagation results obtained with the wild type MMTV LTR vectors and further validated that the intact 5′ UTR is as critical for efficient RNA propagation as it is for successful RNA packaging (Figures 1, 5 and Table 1), but is not sufficient by itself for either of the two processes.

**Table 1:** Propagation efficiency of MMTV transfer vector RNAs containing chimeric 5′ LTRs.

Vector Name	Description of Transfer Vectors Used in Each Transfection	Other Plasmids Added in Each Transfection*	Normalized** CFU/ml ± SD
Mock	No transfer vector	JA10 + MD.G + pGL3	<1
DA19	Entire UTR*** + 30 bp of *gag*	JA10 + MD.G + pGL3	25±6
DA20	Entire UTR +60 bp of *gag*	JA10 + MD.G + pGL3	28±6
DA21	Entire UTR +90 bp of *gag*	JA10 + MD.G + pGL3	133±14
DA22	Entire UTR +120 bp of *gag*	JA10 + MD.G + pGL3	635±48
DA23	Entire UTR +150 bp of *gag*	JA10 + MD.G + pGL3	667±83
DA24	Entire UTR +400 bp of *gag*	JA10 + MD.G + pGL3	1575±207
FA21	PBS only	JA10 + MD.G + pGL3	<1
FA22	32 bp of UTR without *gag*	JA10 + MD.G + pGL3	<1
FA23	64 bp of UTR without *gag*	JA10 + MD.G + pGL3	<1
FA24	96 bp of UTR without *gag*	JA10 + MD.G + pGL3	<1
FA25	128 bp of UTR without *gag*	JA10 + MD.G + pGL3	<1
FA26	Entire UTR without *gag*	JA10 + MD.G + pGL3	<1
NS07	PBS only +400 bp *gag*	JA10 + MD.G + pGL3	<1
NS08	32 bp of UTR +400 bp of *gag*	JA10 + MD.G + pGL3	<1
NS09	64 bp of UTR +400 bp of *gag*	JA10 + MD.G + pGL3	<1
NS10	96 bp of UTR +400 bp of *gag*	JA10 + MD.G + pGL3	<1
NS11	128 bp of UTR +400 bp of *gag*	JA10 + MD.G + pGL3	<1
NS12****	Entire UTR +400 bp of *gag*	JA10 + MD.G + pGL3	403±82

* JA10, MMTV gag/pol packaging expression vector; MD.G, vesicular stomatitis virus (VSV-G) envelope expression vector; pGL3, luciferase expression vector.

** Propagation of the transfer vector RNA expressed as hygromycin resistance colony forming units (CFU)/ml of viral supernatant that was used to infect target cells. The data represents the mean of at least three independent transfection and infection experiments testing all mutants and was derived after normalization to the transfection efficiencies observed by luciferase expression from a co-transfected luciferase expression vector. SD, standard deviation.

*** Entire UTR refers to 160 bp excluding 17 bp of primer binding site (PBS).

**** The differences observed in the RNA propagation abilities of DA24 and NS12, both containing same amounts of 5′ UTR and gag sequences could be attributed to an artificially introduced *Spe*I site in NS12 at the junction of 5′ UTR and gag during cloning. This *Spe*I site may have destabilized some sequence/structural motifs important for MMTV transfer vector RNA packaging and propagation (compare DA24 and NS12 in Figure 5C).

## Discussion

**Figure 6:**
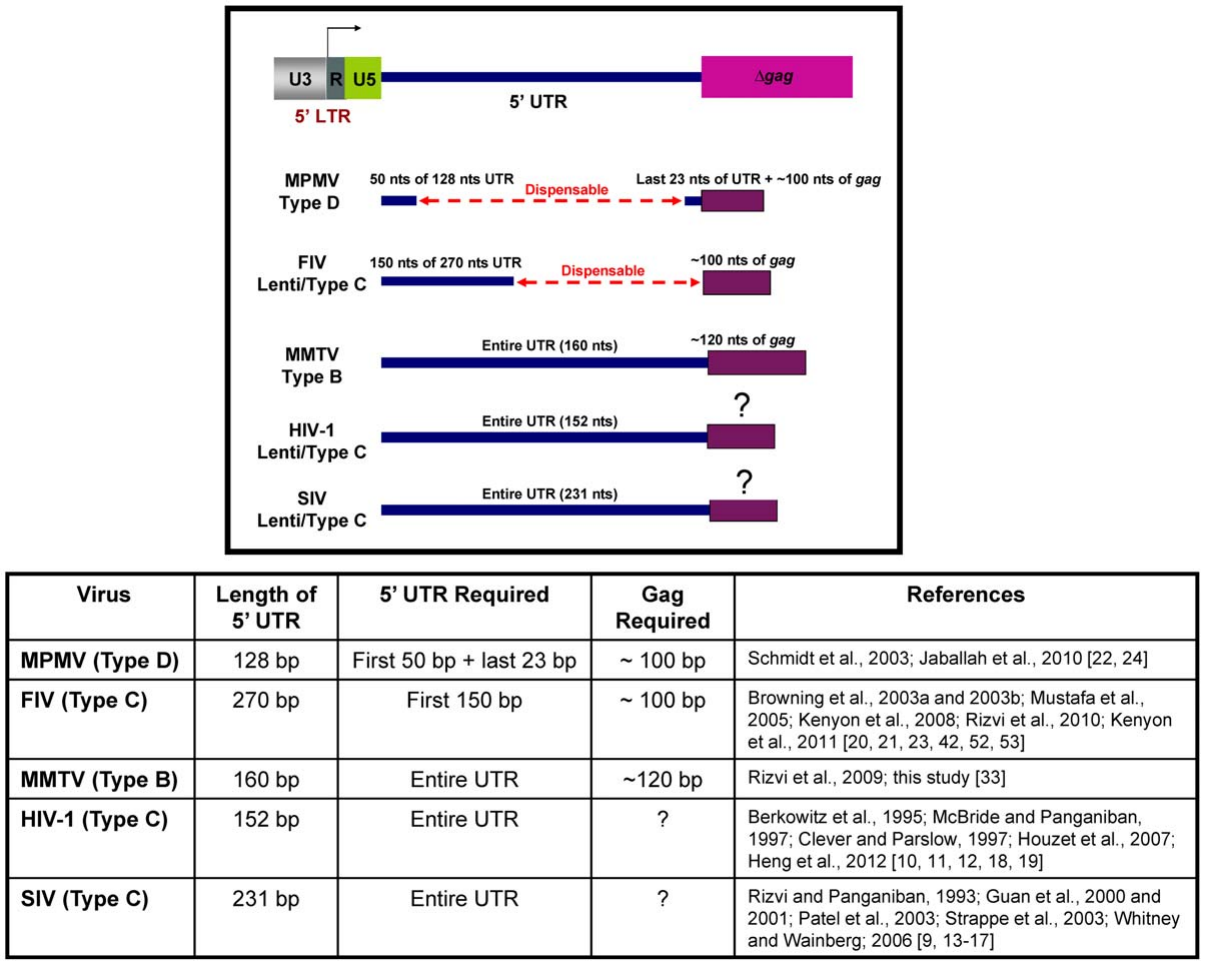
Schematic representation of the packaging determinants observed empirically in different retroviral systems starting from nt +1 in R to the beginning of *gag*. The figure compares the published data pertaining to the requirements of the 5′ UTR and *gag* sequences for optimal RNA packaging in different retroviruses. The table provides further details of the RNA packaging determinants required as observed in various studies referenced in the last column.

It is a well-established fact now that the major determinants of retroviral gRNA packaging lie at the 5′ end of the retroviral genome downstream of R and extending into the *gag* gene (reviewed in [4]–[8]); however, specific residues within this region and their spatial organization may vary among different retroviruses. We have used a similar deletion analysis of the 5′ UTR and *gag* in MPMV [22], [24] and FIV [20]–[23] to determine the precise boundaries of their packaging determinants. Our results have revealed that both MPMV, a simple retrovirus, and FIV, a complex lentivirus, have bipartite packaging determinants composed of regions at the flanks of the 5′ UTR and *gag* sequences tested (Figure 6). These packaging determinants have been shown to assume higher order structures important for RNA packaging and dimerization [24], [42], [52], [53]. This is contrary to what we report here for MMTV where the entire MMTV 5′ UTR was found to be critical for both RNA packaging and propagation. However, our observations with MMTV are similar to what has been observed for HIV-1 and SIV [9]–[19], where most of the 5′ UTR has been shown to be important for packaging of the respective viruses (Figure 6).

The importance of the entire 5′ UTR in retroviral RNA packaging is not surprising and may reflect the presence of *cis* acting sequences/structural motifs important in other steps in viral life cycle as well such as genomic RNA dimerization, reverse transcription, transcriptional activation, nucleocytoplasmic transport of unspliced RNA, and efficiency of RNA translation [1]–[8], [54], [55]. The presence of so many *cis*/structural features with critical functions in a small region may make it difficult to identify a unique or minimal packaging determinant. For example, it has been shown that RNA dimerization and packaging are interlinked phenomenon with one dependent upon the other depending upon the different retrovirus [4]–[8], [56]–[59]. Thus, perturbation of one may affect the other phenomena by default. For instance, the FIV DIS is present within its *gag* gene, a retrovirus in which a bipartite packaging signal has been observed [42], [52], [53], and for this reason parts of the 5′ UTR have been shown to be dispensable for FIV RNA packaging [23]. On the other hand, the HIV-1 DIS is present within the 5′ UTR [60], suggesting the necessity of the presence of the entire UTR for optimal packaging as has recently been shown by Summers and colleagues [19].

Prior to dimerization and packaging, gRNA must be efficiently exported to the cytoplasm and therefore it has been suggested that retroviral gRNA nuclear export, dimerization and packaging mechanisms are also interlinked [6], [61]–[63]. Consistent with this, packaging signal of some retroviruses have been shown to play an important role in the nuclear export of viral gRNA [64], [65]. For example, an internal loop in the packaging signal of HIV-1 closely resembles HIV Rev Responsive Element (RRE) and binds to Rev protein [61], [66]. Mutations in this loop have been shown to reduce nuclear export of viral gRNA and packaging [62]. Similar to HIV-1 Rev/RRE, MMTV also contains a nuclear export pathway (Rem/Rem-Responsive Element (RmRE), facilitating the transport of gRNA, but not partially spliced env RNA [67]–[69]. Furthermore, MMTV RmRE can also assume a higher order structure with multiple loops where its cognate counterpart, Rem, can bind and multimerize to mediate RNA export [37], [38], [68], [69]. Based on their mutual similarity, it has been hypothesized that MMTV may also contain two RmREs; one at the 5′ end (encompassing packaging sequences of MMTV gRNA) present only in unspliced RNA and the second one at the 3′ end present in all MMTV RNAs, facilitating nuclear export of the gRNA and translation of all mRNAs, respectively [69]. The recent study by Hohenadl et al., looking at the effects of 5′ UTR deletions on MMTV env mRNA expression, supports the existence of such an element at the 3′ half of the MMTV 5′ UTR [39]. Deletion of this region in our assay severely impacted RNA packaging (Figures 5C); however, we cannot assess its effect on mRNA transport since all our vector RNAs contained CTE which could have compensated for such transport defects, leading to the efficient export of these vector RNAs to the cytoplasm (Figure 4C). Experiments are currently underway to delineate the overlapping nature of MMTV packaging sequences with those of the recently hypothesized additional nucleocytoplasmic export regulatory element, RmRE.

The present study reveals that the MMTV region starting from R at the 5′ end up to ∼120 bp in the *gag* gene is important for genomic RNA packaging. One of the earliest studies on MMTV RNA packaging tested the role of the 5′ LTR by substituting the 5′ MMTV LTR with that of the Rous sarcoma virus (RSV) LTR [32]. Genomic RNAs encoded by this recombinant virus were defective for RNA packaging despite the fact that they contained the entire 5′ UTR and the remaining viral genome. It is plausible that the impaired packaging observed could have been due to: 1) the role of R/U5 sequences, if any, in packaging in addition to 5′ UTR and *gag* sequences, 2) the insensitivity of the assays used to study RNA packaging. In their assay, RNA from a 100 ml of a wild type virus stock containing 2×10^7^ virions/ml (2×10^9^ virus particles total) was required to observe packaging efficiently on a dot blot assay. In contrast, in our real time-based PCR assay, we could efficiently observe packaged retroviral subgenomic RNAs from vectors that gave rise to even 25 CFU/ml (Figure 5C and Table 1). Since we did not investigate the R/U5 sequences in our mutational analysis, we cannot rule out the role of MMTV R and U5 sequences independently on MMTV RNA packaging (all our constructs contained this region unaltered). It has been well-established that R/U5 can harbor retroviral RNA packaging determinants ([70] and references therein). However, given the observation that our vectors that contained even a 32 nt deletion at the 3′ end of the 5′ UTR (FA25 and NS11, Figures 2B and 5C) were essentially defective for RNA packaging despite containing R/U5 suggests that the role of R/U5 on RNA packaging is not significant at the primary sequence level, though its independent effect as part of a larger secondary structural element, if any, cannot be ruled out.

Interestingly, our similar and systematic deletion analysis of the 5′ UTR and *gag* regions of three different retroviruses, FIV [20], [21], [23], [42], [52], [53], MPMV [22], [24], and MMTV (the present study), has revealed that not only *gag* sequences are required for packaging, but that the amount of *gag* sequences needed are quite similar (100–120 nt) (Figure 6). Considering that 100–120 nt are enough to only code for peptides, it is plausible that the presence of *gag* residues either folds into *cis*-acting structural motif(s) or provide stability to the RNA higher order structure that the 5′ region may assume. Consistent with this, it has recently been shown that at least for FIV and MPMV, the *gag* sequences are involved in long range interaction (LRI) with sequences at the 5′ end of the gRNA [24], [52], [53]. In the case for FIV, any perturbations in the LRI involving the *gag* sequences results in destabilization of the overall RNA secondary structure and consequently, RNA packaging and propagation [42]. The role of *gag* sequences is not so clear in the case of SIV since most of the studies conducted on SIV RNA packaging have maintained *gag* sequences [13]–[17]. However, the same may be true for HIV where even a shorter *gag* region (9 nts) may be required for efficient RNA packaging without affecting RNA dimerization or nucleocapsid binding [19]. These 9 nucleotides have been shown to be involved in maintaining the U5:AUG base pairing important for maintaining the overall RNA secondary structure of the 5′ end of the gRNA [19]. This region may function as a riboswitch, allowing the genomic RNA to switch between a form that promotes translation to a form that promotes RNA dimerization, packaging, and nucleocapsid binding [71]. The structural significance of this region may also explain why the *Spe* I restriction site insertion between the end of 5′ UTR and *gag* AUG had resulted in lower packaging efficiency of the NS12 vector compared to the DA24 (Figure 5C).

Over the years, it is becoming increasingly clear that retroviral RNA packaging and dimerization, regardless of the primary sequence, are dependent on RNA structural motifs of the packaging determinants and their interaction(s) at the structural level (RNA-RNA interactions during dimerization and RNA-protein interactions during packaging) (reviewed in [4]–[8]). This could provide an explanation for the RNA cross- and co-packaging observed among molecularly and phylogenetically divergent retroviruses ([27]–[30] and references therein). Consistent with this, we have recently shown that MMTV and MPMV RNAs could be reciprocally cross-packaged by the heterologous viral particles, suggesting that pseudotyping between two divergent retroviruses can take place at the RNA level [26] and conceivably RNA structural motifs of these viruses facilitate this process. Therefore, it will be important to investigate whether the sequences responsible for augmenting MMTV gRNA packaging assumes any RNA secondary structure that is similar or distinct from other retroviruses, and if so, which particular structural motifs are essential during the MMTV RNA packaging process. Delineation of MMTV gRNA packaging determinants presented in this study should enhance our understanding of MMTV replication, which is imperative if MMTV vectors are to be used successfully for inducible and targeted human gene therapy.

## Materials and Methods

### Plasmid Construction

MMTV packaging construct, JA10, and the transfer vector, DA24, have been derived from HYBMTV, the molecular clone of MMTV [72] and their design and cloning strategies have been described earlier [33]. Briefly, JA10 expresses the MMTV *gag/pol* genes in the presence of the MPMV CTE using the hCMV promoter and the bovine growth hormone (BGH) poly A sequences. The transfer vectors were created as three series: the DA series with incremental deletions of *gag* sequences in the presence of the entire 5′ UTR, the FA series with incremental deletions of the 5′ UTR sequences in the absence of *gag*, and the NS series with incremental deletions of the 5′ UTR sequences in the presence of 400 nt of *gag* (Figures 1B and 2B). Both the vector series with the wild type MMTV LTR (Figure 1B) and the chimeric CMV/RU5 LTR (Figure 2B) were created through several stages of cloning using PCR amplification of the 5′ end of the HYBMTV clone, from the first nucleotide in R to the various regions specified in the constructs using specific primers listed in Table 2. Initially, the three series of transfer vectors maintained the MMTV wild type LTR (Figure 1B); however, due to their low expression in human cells despite hormone induction, the U3 region of the LTR containing the MMTV promoter was replaced by the hCMV promoter via PCR-based cloning, as described earlier [33]. Specific details of the plasmid construction can be obtained from the authors upon request.

**Table 2:** Description of primers used for cloning of the DA, FA, and NS series of vectors, with either the MMTV LTR or the chimeric 5′ LTR containing CMV/R/U5.

Oligo Name	Genomic Location	Size	S/AS*	Sequence (5′ to 3′)	Description
OTR 551	From the start of HYBMTV 5′ U3	28	S	GCATCGATAATGCCGCGCCTGCAGCAGA	2 extra bp/*Cla*I/nt 1–20 of 5′ U3 of HYBMTV
OTR 552	Initial 400 bp of HYBMTV Gag	33	AS	CGACTAGTGATATCGTTCCCCTGGTCCCATAAG	2 extra bp/*Spe*I/*EcoR*V/nt 1885–1867 HYBMTV Gag
OTR 553	HYBMTV Env	40	S	GCACTAGTGCTAGCCCTACATGGTTCTGGGAAAATTCTCC	2 extra bp/*Spe*I/*Nhe*I/nt 7485–7509 HYBMTV Env
OTR 554	End of HYBMTV (at the end of 3′ U5)	29	AS	CAGGGTACCGCTGCCGCAGTCGGCCGACC	3 extra bp/*Kpn*I/nt 9877–9857 HYBMTV 3′ U5 end
OTR 555	HYBMTV 160 bp 5′ UTR (entire 5′ UTR)	28	AS	CGACTAGTTTCCAATGGCTCACCGTAAC	2 extra bp/*Spe*I/nt 1485–1468 of HYBMTV 5′ UTR
OTR 556	HYBMTV 30 bp Gag	28	AS	CGACTAGTTTTCTGCCCTTTTGAGCCCG	2 extra bp/*Spe*I/nt 1515–1495 of HYBMTV Gag
OTR 557	HYBMTV 60 bp Gag	28	AS	CGACTAGTGAGGAGCCTTTGTAAAACAG	2 extra bp/*Spe*I/nt 1544–1525 of HYBMTV Gag
OTR 558	HYBMTV 90 bp Gag	28	AS	CGACTAGTACTCTCTTTCACATGAAGAC	2 extra bp/*Spe*I/nt 1574–1555 of HYBMTV Gag
OTR 559	HYBMTV 120 bp Gag	29	AS	CGACTAGTTATTAGAAACTGATAAAATTC	2 extra bp/*Spe*I/nt 1604–1585 of HYBMTV Gag
OTR 560	HYBMTV 150 bp Gag	28	AS	CGACTAGTTCCTTCTTCGGGAAACCAAG	2 extra bp/*Spe*I/nt 1634–1615 of HYBMTV Gag
OTR 561	HYBMTV PBS only	28	AS	CGACTAGTTCCCTGTTCGGGCGCCAGCT	2 extra bp/*Spe*I/nt 1324–1305 of HYBMTV PBS
OTR 562	HYBMTV 32 bp 5′ UTR	28	AS	CGACTAGTAGAAATAGAGACAAGGGTCA	2 extra bp/*Spe*I/nt 1354–1337of HYBMTV 5′ UTR
OTR 563	HYBMTV 64 bp 5′ UTR	28	AS	CGACTAGTAAAGAGACAATACAAGACAA	2 extra bp/*Spe*I/nt 1388–1369 of HYBMTV 5′ UTR
OTR 564	HYBMTV 96 bp 5′ UTR	28	AS	CGACTAGTCCGTTCCGCTCTTGTGATGA	2 extra bp/*Spe*I/nt 1420–1401 of HYBMTV 5′ UTR
OTR 565	HYBMTV 128 bp 5′ UTR	28	AS	CGACTAGTCGTAGGCGGGACTGCAGCTC	2 extra bp/*Spe*I/nt 1452–1433 of HYBMTV 5′ UTR
OTR 617	Start of HYBMTV 5′ R	35	S	CGCAAGCTTGAGCTCGAGTAAACTTGCAACAGTCC	3 extra bp/*Hind*III site/nt 1163–1182 of HYBMTV R

* S/AS, sense or antisense.

### Transfection and Infection of Cells

The human epithelial kidney cell line, 293T, was used for the production of VSV-G-pseudotyped-MMTV virus particles containing RNA from the various constructs using a modified calcium phosphate method as described [73]. The transfections for each mutant transfer vector were carried out in 6-well plates with 1–2 plates per vector (depending upon the experiment) along with the control luciferase expression vector, pGL3 (Promega, USA), to monitor transfection efficiency as described earlier [23]. Transduction by the marker *hygromycin* resistance gene present on the transfer vector RNA was observed by infection of the human cervical cancer cell line (HeLaT4) by the pseudotyped virus particles and selection for hygromycin-resistant colonies, as described previously [41].

### RNA Isolation, Nucleocytoplasmic Fractionation of Cells and RT PCR

Transfected cells were fractionated into nuclear and cytoplasmic fractions to allow separate analysis of RNA residing within the two cellular compartments as described earlier [23]. RNA from each fraction was isolated from transfected cells using the Trizol reagent, as per manufacturer's directions (Invitrogen, USA). Integrity of the nucleocytoplasmic fractionation was monitored by the absence of the nuclear-only unspliced ß-actin mRNA in the cytoplasmic RNA fractions by reverse transcriptase PCR (RT-PCR). Briefly, following DNase treatment, equal RNA amounts (2.5 ug per sample for each fractions) were tested for DNA contamination by PCR amplification for 30 cycles using primers specific for a house keeping gene, glyceraldehyde-3-phosphate dehydrogenase (GAPDH) (Figure 2C, panel III). Lack of any amplifiable signal confirmed that the RNA preparations were not contaminated with DNA carried over from the transfected cultures. Next, the DNased RNA fractions were reverse transcribed and amplified using primers specific for unspliced and spliced ß-actin mRNA to confirm whether nuclear membrane integrity was maintained during fractionation or not. As an additional control, multiplex amplifications were performed in the presence of primers/competimer for 18S ribosomal RNA, ensuring that each sample in the unspliced ß-actin PCRs contained amplifiable cDNAs.

### Southern Blot Analysis

Some of the packaged viral RNA samples were subjected to Southern blot analysis as described earlier [70] for which the probe was prepared by PCR amplification using primers within the 5′ R/U5/PBS region common to all the transfer vectors as follows: OTR671 (+) GTC CTA ATA TTC ACG TCT CGT GTG, nt 1179–1202 and OTR 672 (−) CTG TTC GGG CGC CAG CTG CCG CAG, nt 1298–1321. Hybridization was performed using the AlkPhos Direct Labeling Kit (Amersham, USA) as per manufacturer's directions.

### Real Time PCR Analysis of Transfer Vector Expression

To test the relative levels of transfer vector RNAs expressed within the cytoplasm and packaged into the viral particles, a real time PCR custom expression assay was developed using the minor groove binding (MGB)/FAM and MGB/VIC chemistry from Applied Biosystems, USA. Additionally, the ABI real time PCR assays use ROX as an internal fluorescence reference dye to which the target reporter dye signal (either FAM or VIC, in our case) is normalized to during data analysis, to correct for fluorescence fluctuations caused by changes in concentration or volume. The PCR primers were designed to anneal within a region at the beginning of the MMTV U5 region (nt 1192–1259), a 68 nt region common to all the constructs, enabling their relative quantitative assessment using the same probe/primer combination concomitantly. The sequence of the probe and primers were as follows: Probe (MTV-1LTR-SITEM1 FAM): TCGCCATCCCGTCTCC (16-mer; nt 1214–1229); Forward primer (MTV-1LTR-SITEF): CGTCTCGTGTGTTTGTGTTTGTGTCTGT (22-mer; nt 1192–1213); Reverse primer (MTV-1LTR-SITER): CCTCTGGAAAGTGAAGGATAAGTGA (25-mer; nt 1259-1235). The assay was validated *in silico* using the Primer Express tool as well as the ABI design pipeline bioinformatic tools that ensure specificity, reproducibility, and amplification efficiency of the assay performance. Further validation of the PCR amplification efficiency was conducted by testing the assay using the online web tool “pcrEfficiency” [74] that confirmed it to be over 100%. As an endogenous control, the optimized ß-actin MGB VIC-labeled assay (no 4326315E with limited primer concentration) from ABI was used (Applied Biosystems, USA).

To determine which method to use for the Relative Quantification analysis, the standard curve or the comparative (ΔΔCt) method, a 10- and serial 2-fold dilutions of a cDNA prepared from 293T cells expressing DA24 were tested in non-multiplex RT-PCRs in triplicates assuming conservatively that 1 ug of input RNA results in ∼100 ng of single stranded cDNA using random hexamers as primers and MMLV RT (Promega; Figure 3A and 3B). The resultant Ct values were plotted on a scatter plot against the pg of cDNA tested in each dilution in triplicates to generate standard curves for both assays (Figure 3C and 3D). This was followed by calculating the ΔCt values (Ct values from MMTV assay – Ct value of ß-actin endogenous control) which were plotted against the input cDNA to determine the slope of the curve (Figure 3E).

For the final relative quantification analysis, each viral RNA sample was tested in triplicates in multiple assays using the Taqman Universal Master Mix (Applied Biosystems and the 7500 Real Time PCR System (Applied Biosystems, USA), while the cytoplasmic RNA was tested in duplicates. As mentioned earlier for the cytoplasmic RNA fractions, 1/5^th^ of the total viral RNA preparations were first DNase-treated, confirmed for the absence of DNA by PCR using vector RNA specific primers, and then reverse transcribed into cDNA, as previously described [73]. Equal amounts of the resulting cDNAs were tested for vector expression in the cytoplasmic fractions as well as their packaging in the viral particles using the real time PCR assay and the following cycling conditions: an initial denaturation step of 10 minutes at 94°C followed by 40 cycles of denaturation and annealing/extension steps at 95°C for 15 secs and 60°C for 1 min. The relative packaging efficiency for each mutant transfer vector RNA was determined after normalization of the data with the endogenous control, ß-actin, as well as the control for transfection efficiency, luciferase expression.

### Statistical Analysis

To determine whether the observed differences in the normalized packaging efficiencies were statistically significant, a standard, paired, two-tailed Student's *t*test was performed between various constructs using the Microsoft Excel software. A value of p<0.01 was considered statistically significant.
